# Organised Pleural Haematoma Causing Left Ventricular Compression and Tamponade-Like Pathophysiology

**DOI:** 10.7759/cureus.114646

**Published:** 2026-08-17

**Authors:** Takudzwa J Dhlandhlara, Fahmida Begum, Rachna Awasthi, Vijayan Suresh, Rahul Mukherjee

**Affiliations:** 1 Renal Medicine, Heartlands Hospital, Birmingham, GBR; 2 Respiratory Medicine, Heartlands Hospital, Birmingham, GBR; 3 Pathology, Queen Elizabeth Hospital Birmingham, Birmingham, GBR

**Keywords:** computed tomography, echocardiography, extracardiac compression, left ventricular compression, pleural haematoma

## Abstract

Pleural haematomas are rare and may arise following trauma or supratherapeutic anticoagulation. Chronic organised pleural haematomas pose diagnostic and therapeutic challenges due to their mass-like appearance and resistance to drainage. We report the case of a man in his 50s with end-stage renal disease on haemodialysis who developed progressive dyspnoea and hypotension several years after a persistent pleural effusion. Computed tomography demonstrated a pleural-based mass suspicious for malignancy, while transthoracic echocardiography revealed preserved systolic function with external compression of the left ventricular lateral wall and elevated filling pressures. An initial image-guided biopsy was inconclusive; however, subsequent surgical exploration identified an organised pleural mass containing old blood. Histology confirmed an organised haematoma with no evidence of malignancy. Unfortunately, the patient died before definitive surgical intervention. This case highlights an unusual cause of extracardiac compression mimicking malignancy and producing cardiac tamponade-like pathophysiology in the absence of pericardial disease. Early recognition using multimodal imaging is essential to prevent delays in management and adverse outcomes.

## Introduction

Pleural haematomas are uncommon and result from the accumulation of blood within the pleural space. The existing literature more commonly describes extrapleural haematomas, which typically occur in the setting of trauma or supratherapeutic anticoagulation. However, extrapleural haematomas represent a distinct clinical entity from pleural haematomas, referring to an abnormal accumulation of blood between the parietal pleura and the endothoracic fascia [1-3].

Over time, retained haemorrhagic collections may undergo organisation into encapsulated masses, mimicking pleural neoplasms or loculated effusions, and can become refractory to conventional drainage due to their near-solid consistency. Large collections can exert significant mass effect, leading to respiratory compromise, mediastinal shift, and, in severe cases, circulatory disturbance due to cardiac compression [1].

Pleural effusions have been reported to produce tamponade-like pathophysiology through impaired cardiac filling [4]. To the best of our knowledge, direct left ventricular compression resulting from an organised pleural haematoma is exceedingly rare, with no identical cases identified in our search of PubMed and MEDLINE.

We present a case of an organised pleural haematoma causing left ventricular compression, highlighting the diagnostic challenges and potential for significant haemodynamic compromise.

## Case presentation

A man in his 50s presented in January 2019 with persistent mild dyspnoea over several weeks. His medical history included hypertension and end-stage renal disease secondary to IgA nephropathy, requiring maintenance haemodialysis initially via a right-sided tunnelled dialysis catheter and then later via a right-sided femoral arteriovenous graft. He had previously undergone cadaveric renal transplantation in 2012, which failed six years later. His long-term medications did not include antiplatelets or anticoagulant therapy.

**Figure 1:**
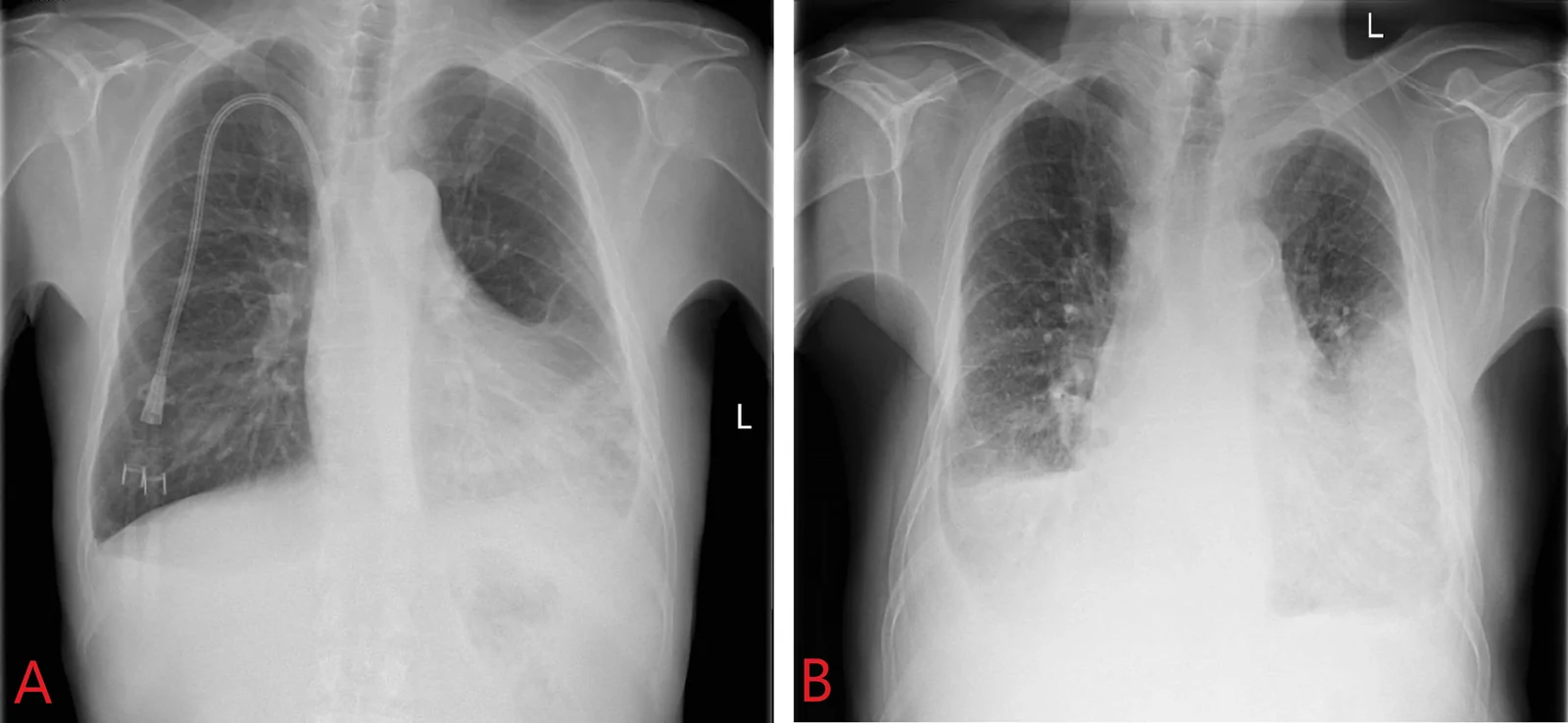
Chest radiographs. (A) January 2019 (A) and (B) July 2025, showing persistent left-sided mid-to-lower zone opacification, which is representative of the organised pleural haematoma.

A chest radiograph done at presentation (Figure 1A) identified a left-sided pleural effusion. Pleural fluid analysis was exudative (protein: 45 g/L) with no malignant cells on cytology, with normal pH and triglyceride levels. The rest of his laboratory investigations were as indicated in Table 1. He had three unsuccessful therapeutic pleural drains. As he had no functional limitation, there were no further thoracocentesis attempts and the pleural effusion was managed conservatively with surveillance.

**Table 1:** Laboratory investigations in January 2019, June 2022, July 2025, and September 2025. Hb: Haemoglobin, WCC: White Cell Count, INR: International Normalised Ratio, eGFR: Estimated Glomerular Filtration Rate, CRP: C-Reactive Protein, LDH: Lactate Dehydrogenase, mcs: Microscopy, Culture and Sensitivity, HBsAg: Hepatitis B Surface Antigen, Ab: Antibody

Test Name	Reference Range	January 2019	June 2022	July 2025	September 2025
Haematology					
Hb (g/L)	130-171	72	87	90	99
WCC (x10^9^/L)	3.26-11.20	7.94	5.21	5.98	8.25
Platelet count (x10^9^/L)	150-400	103	99	120	128
INR (mm/hr)	-	1.0	1.1	1.0	1.0
Biochemistry					
Sodium (mmol/L)	133-146	142	135	134	137
Potassium (mmol/L)	3.5-5.3	5.6	5.4	4.7	4.5
Urea (mmol/L)	2.5-7.8	18.5	21.8	16.4	14.6
Creatinine (mmol/L)	64-104	599	585	621	512
eGFR (mL/min/1.73 m^2^)	>60	9	9	8	10
CRP (mg/L)	<4	18	9	10	36
Pleural fluid protein (g/L)	-	45	-	-	-
Pleural fluid LDH (U/L)	-	149	-	-	-
Pleural fluid triglycerides (mmol/L)	<0.56	0.25	-	-	-
Pleural fluid pH	-	7.33	-	-	-
Microbiology					
Pleural fluid mcs	-	No growth	-	-	-
Pleural fluid cytology	-	Mesothelial cells are present together with some lymphocytes. No malignant cells are seen.	-	-	-
HIV 1 & 2 serology	-	Negative	-	-	-
Syphilis serology	-	Negative	-	-	-
HBsAg	-	Negative	-	-	-
Hepatitis C Ab	-	Negative	-	-	-

Between January 2019 and December 2024, he had been only mildly dyspnoeic without functional compromise. In March 2025, his dyspnoea had progressively worsened. Optimisation of fluid balance and increased ultrafiltration during dialysis were largely ineffective in addressing the dyspnoea. By June 2025, his exercise tolerance had deteriorated significantly such that he was forced into early retirement from his job as a roofer. Despite the significantly functionally limiting shortness of breath, he did not require long-term oxygen therapy. He had also developed gradual hypotension by this time, necessitating discontinuation of his antihypertensive medications.

**Figure 2:**
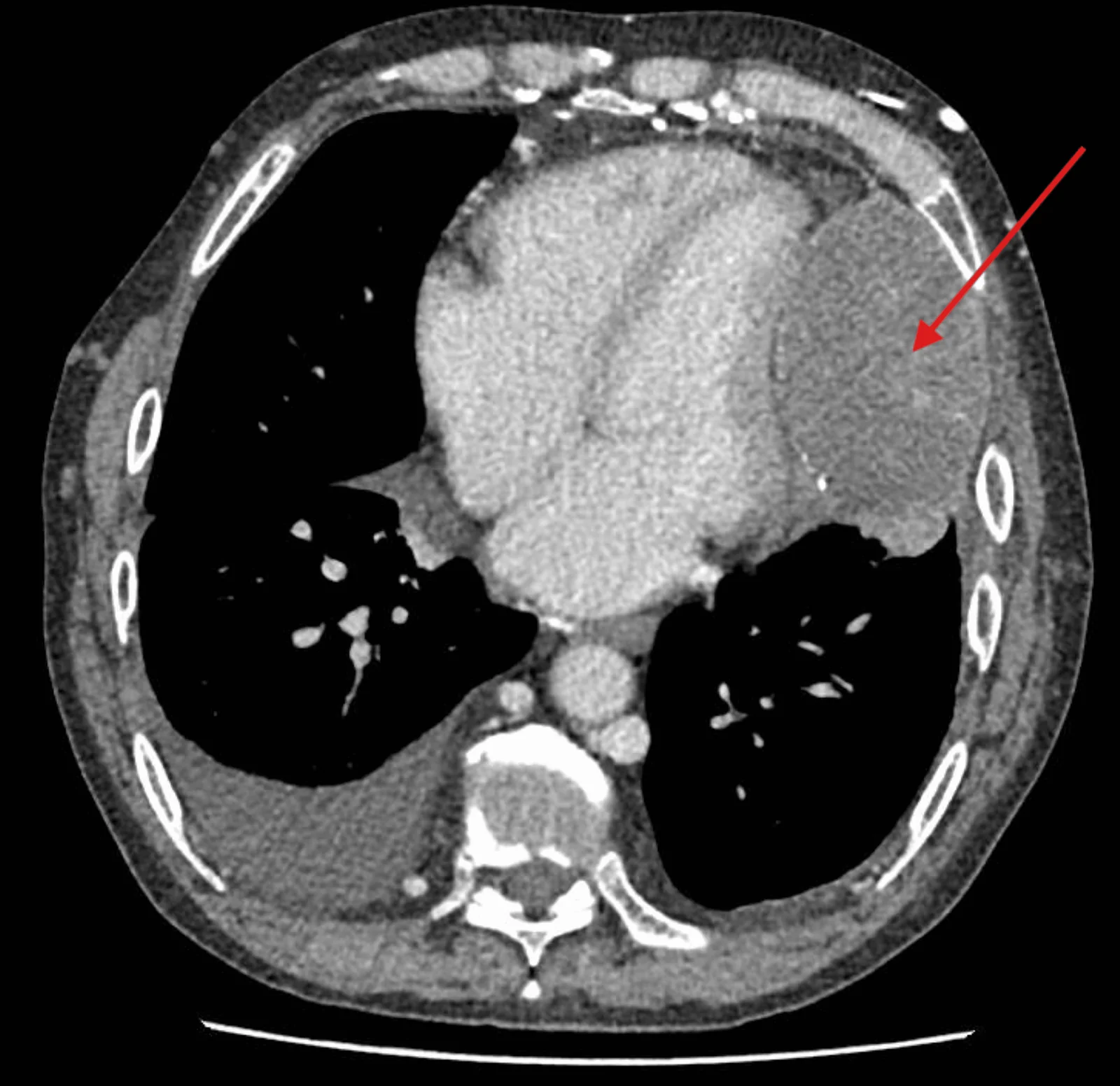
CT image showing the pleural haematoma (red arrow) causing compression on the left ventricle.

**Figure 3:**
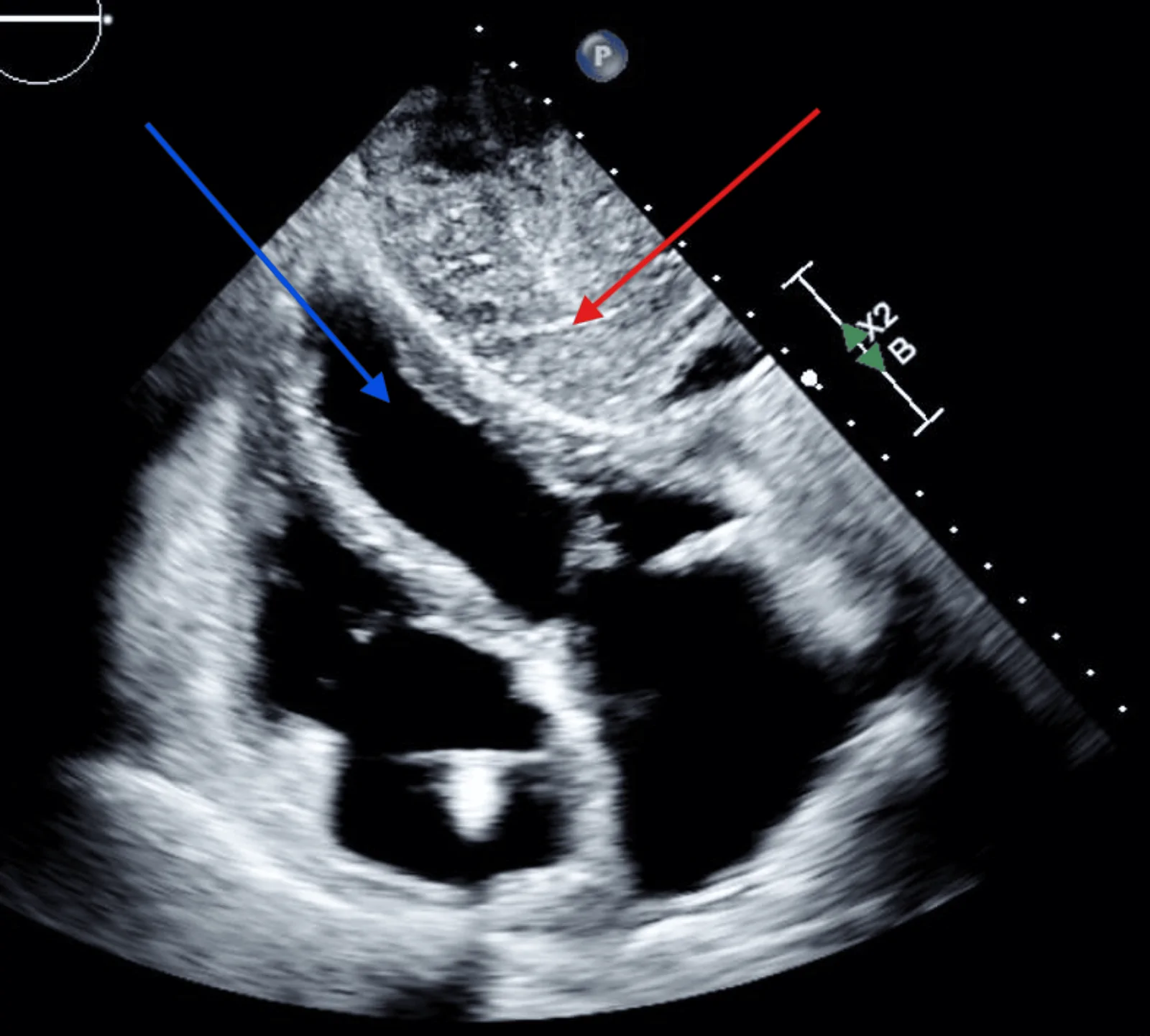
Echocardiogram in diastole showing the left ventricle (blue arrow) and the pleural haematoma (red arrow).

**Figure 4:**
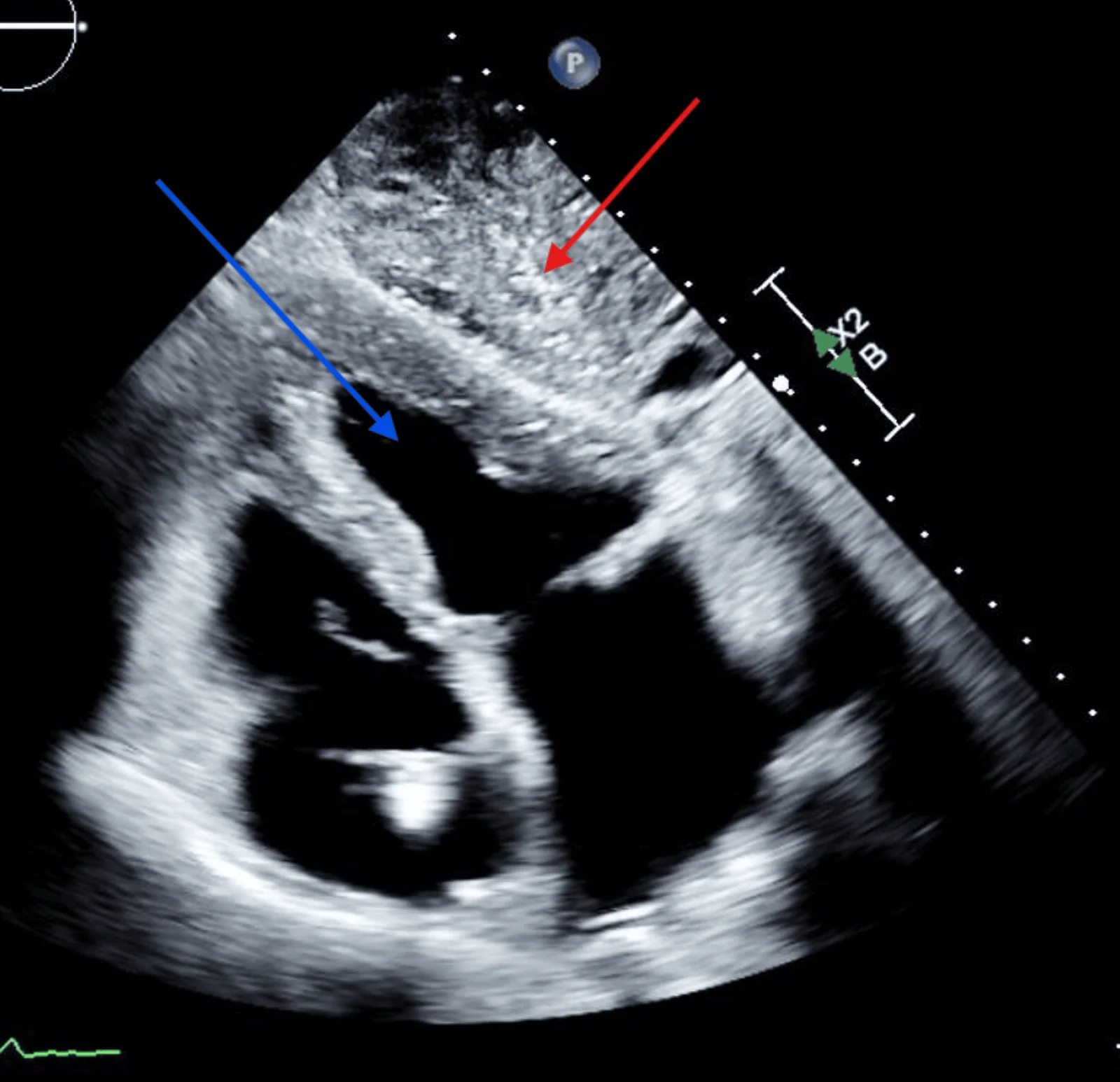
Echocardiogram in systole showing left ventricle (blue arrow) and pleural haematoma (red arrow).

In July 2025, he had several radiological investigations (Figure 1B, Figures 2-4). Chest radiography demonstrated persistent left-sided mid-to-lower zone opacification (Figure 1B). Computed tomography (CT) of the thorax revealed a heterogeneous pleural-based mass measuring 8 cm transversely x 9 cm anterioposteriorly x 14 cm craniocaudally, suspicious for malignancy, causing compression of the left ventricle (Figure 2). 

Transthoracic echocardiography demonstrated preserved ejection fraction (60 ± 5%) and indeterminate diastolic function. There was external compression of the left ventricular lateral wall, elevated filling pressures, and left atrial dilatation (left atrial volume index 55 mL/m^2^; normal <34 mL/m^2^). Right ventricular size and pressures were normal, with no significant valvular disease (Figures 3-4).

**Figure 5:**
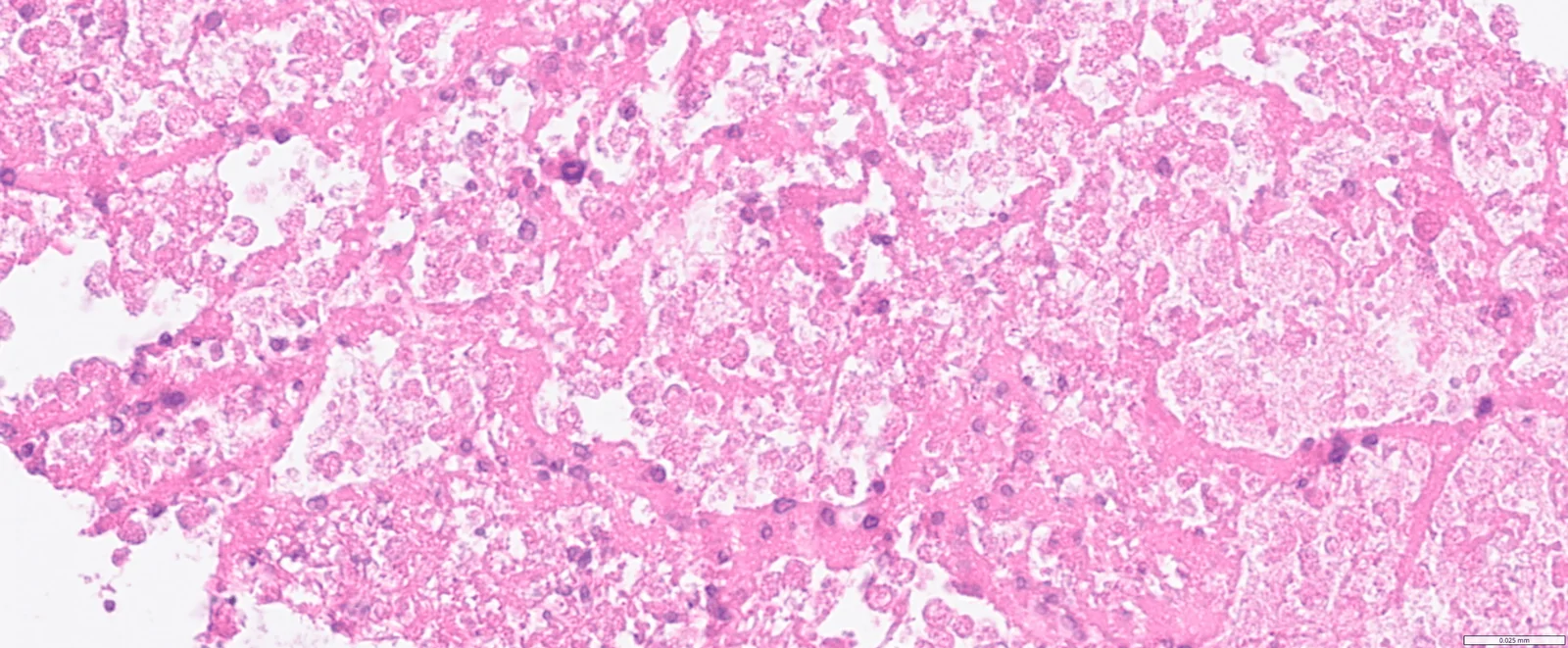
CT-guided needle biopsy of an intrathoracic mass showing predominantly fibrin and some leucocytes (H&E stain, x60). H&E: Haematoxylin & eosin

**Figure 6:**
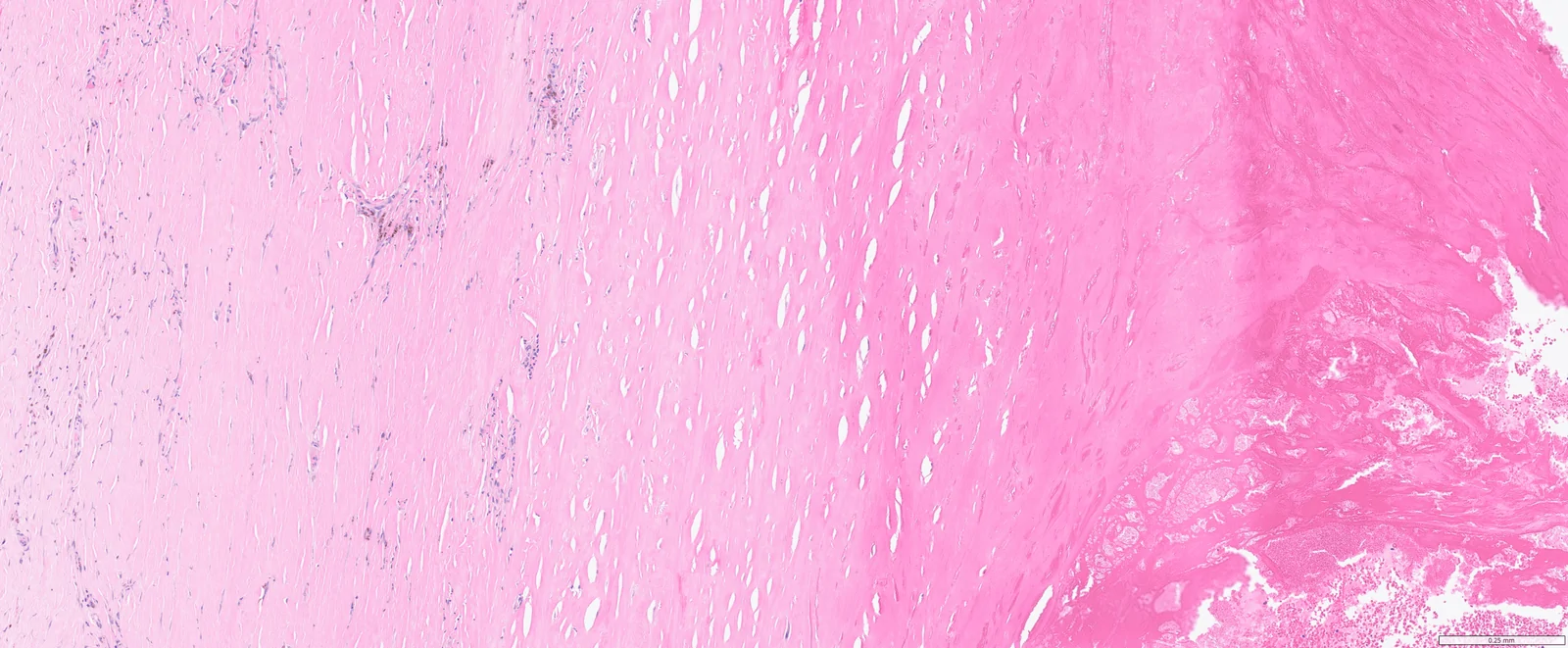
VATS-guided biopsy of an intrathoracic mass showing the fibrous wall with haemosiderin laden macrophages with central haematoma (H&E stain, x40). H&E: Haematoxylin & eosin; VATS: Video-assisted thoracoscopic surgery

In August 2025, he had an outpatient CT-guided needle biopsy, which demonstrated predominantly fibrinous material and was inconclusive for malignancy (Figure 5). The patient subsequently underwent video-assisted thoracoscopic surgery (VATS)-guided biopsy in late September 2025, which revealed an organised pleural mass containing old blood (Figure 6). Histological examination confirmed an organised haematoma with inflammatory exudate and benign reactive fibrofatty tissue, with no evidence of malignancy.

In mid October 2025, while awaiting a definitive surgical management plan, he suffered an out-of-hospital cardiac arrest with unsuccessful cardiopulmonary resuscitation, and he subsequently died. No autopsy was done, and the cause of death, as determined from the clinical history, was cardiogenic shock from a compressive left-sided organised pleural haematoma.

## Discussion

Pleural haematomas most commonly arise following thoracic trauma due to injury to intercostal or internal mammary vessels, often in association with rib fractures [1]. Delayed presentations may occur, particularly in elderly or anticoagulated patients, due to slow accumulation or under-recognition on initial imaging [5]. Organised haematomas frequently represent missed or inadequately treated initial events.

While cardiac tamponade is classically associated with pericardial fluid accumulation, extracardiac pathology may similarly impair cardiac filling and mimic tamponade [6]. Large pleural collections can compress adjacent cardiac chambers, most commonly the left atrium, leading to diastolic dysfunction [7]. Direct left ventricular compression, as observed in this case, is rare and represents a distinct pathophysiological mechanism. Reduced ventricular compliance and impaired diastolic filling may result in decreased stroke volume and haemodynamic instability [8]. Due to the compressive effect of the pleural haematoma, our patient had tamponade-like haemodynamic features, which included gradual hypotension and elevated left ventricular filling pressures.

Chronic organised pleural haematomas differ from acute haemothorax, as retained blood undergoes fibrosis and encapsulation, forming a semi-solid mass that is not amenable to simple drainage. This results in a persistent space-occupying lesion exerting prolonged compressive effects on pulmonary and mediastinal structures.

Diagnosis is often challenging due to non-specific imaging findings. Pleural haematomas may appear as homogeneous opacities or mass-like lesions on chest radiography, mimicking malignancy or pleural effusion [9,10]. CT provides anatomical delineation, while echocardiography is essential for assessing cardiac compression and haemodynamic effects [8]. Histological confirmation is required to exclude malignancy [11].

Intervention is indicated in cases of haemodynamic compromise, progressive symptoms, or significant mass effect. Definitive management typically involves surgical evacuation via VATS or thoracotomy [12,13].

If untreated, pleural haematomas may result in significant complications. Respiratory compromise may occur due to lung compression and atelectasis, leading to ventilation-perfusion mismatch [14]. Cardiovascular compromise may arise from mediastinal shift or direct cardiac compression, potentially mimicking tamponade physiology or leading to heart failure [15]. Chronic complications, including fibrothorax and empyema, further increase morbidity [16].

## Conclusions

Pleural haematoma should be considered in patients with persistent unilateral thoracic opacification, particularly when drainage is unsuccessful and there is progressive cardiorespiratory compromise. Recognition that pleural collections can produce tamponade-like pathophysiology in the absence of pericardial disease is essential.

Early use of CT and echocardiography enables accurate diagnosis, and timely surgical intervention is often required in organised cases to prevent adverse outcomes.
